# Risk factors of 30-day and long-term mortality and outcomes in open repair of thoracoabdominal aortic aneurysm

**DOI:** 10.1186/s13019-024-02666-2

**Published:** 2024-04-02

**Authors:** Sudena Wang, Chunrong Wang, Yuchen Gao, Yu Tian, Jia Liu, Yuefu Wang

**Affiliations:** 1 Department of Anesthesiology, Fuwai Hospital, Chinese Academy of Medical Sciences, Peking Union Medical College, Beijing, China; 2 Department of Surgery Intensive Care Unit, Beijing Shijitan Hospital, Capital Medical University, Beijing, China

**Keywords:** Open repair of descending thoracic and abdominal aortic aneurysm, Mortality, Risk factors, Mortality

## Abstract

**Background:**

Open repair of thoracoabdominal aortic aneurysm (TAAA) was characterized by significant risk of postoperative mortality and morbidity. The aim of this study was to determine the perioperative predictors of early and long-term mortality in patients undergoing open repair of TAAA. Besides, the postoperative outcomes in patients with open repair of TAAA were described.

**Methods:**

This is a single-center retrospective study, and 146 patients with open repair of TAAA from January 4, 2011, to November 22, 2018 was involved. Categorical variables were analyzed by the Chi-square test or Fisher’s exact test, and continuous variables were analyzed by the independent sample t-test and the WilCoxon rank-sum test. Multivariate Logistic regression and Cox regression were applied to identify the predictors of 30-day and long-term mortality, respectively. The Kaplan Meier curves were used to illustrate survival with the Log-rank test.

**Results:**

The 30-day mortality was 9.59% (*n* = 14). Older than 50 years, the intraoperative volume of red blood cell (RBC) and epinephrine use were independently associated with postoperative 30-day mortality in open repair of TAAA. Long-term mortality was 17.12% (*n* = 25) (median of 3.5 years (IQR = 2–5 years) of follow-up). Prior open thoracoabdominal aortic aneurysm (TAAA) repair, aortic cross-clamping (ACC) time, intraoperative volume of RBC and use of epinephrine were independently correlated with long-term mortality.

**Conclusions:**

Identifying perioperative risk factors of early and long-term mortaliy is crucial for surgeons. Intraoperative volume of RBC and use of epinephrine were predictors of both early and long-term mortality. In addition, patients of advanced age, prior open repair of TAAA and prolonged ACC time should be paid more attention.

## Background

The natural progression of thoracoabdominal aortic aneurysm (TAAA) can lead to the aneurysm growing, and finally the aneurysm is prone to rupture without surgery. The prognosis of such patients with TAAA is poor [1, 2]. It is reported that the 5-year survival rate of such patients without surgery is from 7% to19.2% [3]. Currently, the main surgery of TAAA is open repair of thoracoabdominal aortic aneurysm (TAAAR), which can effectively prevent aneurysm rupture. Recently, a systematic review illustrated that the perioperative mortality of 47 studies related to open TAAAR is 8.9% [4]. However, many studies reported that the incidence of mortality and severe complications including permanent spinal cord injury (SCI), renal failure, stroke, myocardial infarction after the TAAAR cannot be ignored [5–7], especially in extent II and III [8]. This research mainly analyses perioperative risk factors of 30-day and long-term mortality in open repair of thoracoabdominal aortic aneurysm  (TAAA, extent II/III).

## Methods

This is a single-center retrospective study including 146 consecutive patients who underwent open repair of TAAA from January 4, 2011, to November 22, 2018. Ethical consent was approved by the Ethics Committee of Fuwai Hospital (Approval NO. 2019 − 1308). The study protocol was compliant with the Declaration of Helsinki. Patient consent was waived.

### Data resource

Patients’ data was collected from electronic medical record system. Clinical data included preoperative basic characteristics, intraoperative variables, postoperative 30-day mortality and adverse complications. In addition to collecting furthermore data, the occurrence and time of mortality and adverse outcomes were followed by our research staff by telephoning patients and patients’ family members in 2020.

### Surgical methods

Under general anesthesia and normothermic conditions with a nasal temperature > 35 °C, a left lateral thoracoabdominal incision was made, involving rib dissection, extending through the left rectus abdominis to the level of the umbilicus. During the procedure, moderate doses of heparin (200 U/kg) was used. The thoracic and abdominal aorta were exposed, and the proximal thoracic aorta was mobilized. A quadrifurcated graft was used to replace the descending aortic aneurysm. One branch of the graft was anastomosed to the severed end of the left external iliac artery, while another branch was connected to a vein for blood return using a blood pump technique. Temporary cross-clamping was applied to thoracic aorta, and proximal end of the synthetic graft was was anastomosed to the severed end of the thoracic aorta and reconstruction of intercostal arteries was performed. The celiac trunk, superior mesenteric artery, and right renal artery were modified into an island configuration, anastomosed to the distant proximal end of the synthetic graft. The left renal artery and bilateral common iliac arteries were anastomosed to branch synthetic grafts. Finally, protamine sulfate was utilized to neutralize heparin. For cases where occlusion of the proximal end of the thoracic aorta was not feasible, surgery was performed under deep hypothermic circulatory arrest (DHCA).

TAAA repair with DHCA involved the administration of heparin (400 U/kg). Initiation of hypothermic circulatory arrest was followed by establishing vena cava drainage through cannulation of the left external iliac vein. Insertion of an arterial cannula into the left external iliac artery facilitated drainage from the left heart, achieved by inserting a tube either into the apical incision or the main pulmonary artery to establish cardiopulmonary bypass. Nasal and rectal temperatures were gradually reduced to 20 °C.

### Definitions

Regarding postoperative complications, hypohepatia was identified as alanine aminotransferase (ALT) > 200 u/L or total bilirubin (TBil) > 50 umol/L or lactic dehydrogenase (LDH) > 500 u/L. Postoperative renal failure was defined as the need for dialysis or hemofiltration as well as renal insufficiency was defined as the serum creatinine level two times than the preoperative baseline value [9]. Postoperative respiratory complications were defined as one or more episodes of postoperative pneumonia, respiratory failure, acute respiratory distress syndrome (ARDS), or pulmonary embolism. SCI was classified, according to its severity, into paraplegia (muscle strength 0 ∼ 2 grade) and paraparesis (muscle strength 3 ∼ 4 grade). In order to provide more guidance for clinical practice, the age was divided into two categories by interquartile range (IQR): 50 (17–70) years. The cut-off point of body mass index (BMI) was 30 kg/m2 on account of the World Health Organization’s BMI diagnosis of obesity [10].

### Outcomes of interest

The primary endpoint was 30-day and long-term mortality. Secondary endpoint was a composite endpoint which included 30-day and long-term adverse outcomes.

### Statistical analysis

Data were analyzed with IBM SPSS Statistics 26.0. Discrete variables and continuous variables were presented as n (%) and mean ± standard deviation, respectively. We tried to find out influencing factors for the mortality in the short term after surgery. Categorical variables were analyzed by using the Chi-square test or Fisher’s exact test. The independent sample t-test and the WilCoxon rank-sum test were used for normally and non-normally distributed continuous variables, respectively. Univariate Logistic regression was performed between groups with and without death within 30 days to select risk factors from all relevant preoperative and intraoperative variables, and each variables showing *P* < 0.05 were entered into multivariate Logistic regression models (method: LR forward) to identify independent risk factors.

Simultaneously, the multivariate Cox regression (method: LR forward) was performed to identify independent risk factors of long-term mortality. For the independent risk factors identifified by Cox regression, the actuarial survival was shown by the Kaplan Meier analysis and differences between the two groups was determined by the Log-rank test. Discrete variables were transformed into categorical variables according to their quartiles as a cut point. P value less than 0.05 was considered significant.

## Results

Of 146 patients undergoing open repair of TAAA, 101 cases were Crawford extent II and 45 cases were Crawford extent III. The average age of study participants was 41.27 ± 11.61 years. The proportion of males (76.03%) was greater. The mean ACC time was 22.29 ± 24.04 min. Preoperative and intraoperative patients characteristics were listed in Table 1.

**Table 1:** Preoperative and intraoperative variables in 146 patients undergoing open repair of TAAA.

Variables	Total (%)		30-day mortality (%)			Long-term death (%)		
			Yes	No	P value	Yes	No	P value
**Preoperative variables**								
Age ≥ 50 (years)		36 (24.66)	7 (4.79)	29 (19.86)	0.047	10 (6.85)	26 (17.81)	0.051
BMI ≥ 30 (Kg/m^2^)		13 (8.90)	0 (0)	13 (8.90)	0.461	1 (0.68)	12 (8.22)	0.575
Male		111 (76.03)	13 (8.90)	98 (67.12)	0.222	19 (13.01)	92 (63.01)	0.997
Current smoking		59 (40.41)	5 (3.42)	54 (36.99)	0.706	12 (8.22)	47 (32.19)	0.396
Hypertension		80 (54.79)	9 (6.16)	71 (48.63)	0.453	18 (12.33)	62 (42.47)	0.058
Peripheral vascular disease		14 (9.59)	0 (0)	14 (9.59)	0.421	1 (0.68)	13 (8.90)	0.503
Prior cardiac surgery		30 (20.55)	1 (0.68)	29 (19.86)	0.338	4 (2.74)	26 (17.81)	0.536
Prior TEVAR		24 (16.44)	3 (2.05)	21 (14.38)	0.880	5 (3.42)	19 (13.01)	0.817
Prior open repair of TAAAR		3 (2.05)	0 (0)	3 (2.05)	1.000	2 (1.37)	1 (0.68)	0.076
Marfan syndrome		39 (26.71)	5 (3.42)	34 (23.29)	0.629	7 (4.79)	32 (21.92)	0.873
Aortic aneurysm		134 (91.78)	11 (7.53)	123 (84.25)	0.167	22 (15.07)	112 (76.71)	0.722
Aortic dissection		107 (73.29)	11 (7.53)	96 (65.75)	0.879	18 (12.33)	89 (60.96)	0.873
CAD		5 (3.42)	1 (0.68)	4 (2.74)	0.400	2 (1.37)	3 (2.05)	0.203
Stroke		6 (4.11)	0 (0)	6 (4.11)	1.000	0 (0)	6 (4.11)	0.559
Chronic renal insufficiency		22 (15.07)	0 (0)	22 (15.07)	0.206	3 (2.05)	19 (13.01)	0.870
Carotid artery diseases		17 (11.64)	5 (3.42)	12 (8.22)	0.012	5 (3.42)	12 (8.22)	0.276
Rupture		3 (2.05)	1 (0.68)	2 (1.37)	0.263	1 (0.68)	2 (1.37)	0.433
ASA					0.822			0.478
Class I, II and III		113 (77.40)	10 (6.85)	103 (70.55)		18 (12.33)	95 (65.07)	
Class IV		33 (22.60)	4 (2.74)	29 (19.86)		7 (4.79)	26 (17.81)	
NYHA					0.216			0.833
Class I and II		133 (91.10)	11 (7.53)	122 (83.56)		22 (15.07)	111 (76.03)	
Class III and IV		13 (8.90)	3 (2.05)	10 (6.85)		3 (2.05)	10 (6.85)	
Maximum aneurysm diameter (cm)		5.83 ± 2.24	4.68 ± 2.65	5.95 ± 2.17	0.105	5.58 ± 2.54	5.88 ± 2.18	0.637
Hemoglobin<120 (g/L)		16 (10.96)	2 (1.37)	14 (9.59)	1.000	3 (2.05)	13 (8.90)	1.000
RBC<3.5 (10^12^/L)		7 (4.79)	1 (0.68)	6 (4.11)	0.514	2 (1.37)	5 (3.42)	0.757
Albumin<35 (g/L)		14 (9.59)	2 (1.37)	12 (8.22)	0.880	3 (2.05)	11 (7.53)	0.939
HCT<0.35 (%)		39 (26.71)	5 (3.42)	34 (23.29)	0.629	7 (4.79)	32 (21.92)	0.873
**Intraoperative variables**								
Non-elective surgery		6 (4.11)	1 (0.68)	5(3.42)	0.460	2 (1.37)	4 (2.74)	0.601
Preoperative CSFD		41 (28.08)	4 (2.74)	37 (25.34)	1.000	7 (4.79)	34 (23.29)	0.992
Fluid volume (ml)		2836.07 ± 1586.00	4463.21 ± 2583.95	2663.49 ± 1344.87	0.014	3763.40 ± 2337.18	2644.47 ± 1315.59	0.069
ACC time (min)		22.29 ± 24.04	33.86 ± 33.84	21.06 ± 22.59	0.434	34.88 ± 32.75	19.69 ± 21.07	0.041
DHCA		7 (4.79)	2 (1.37)	5 (3.42)	0.136	3 (2.05)	4 (2.74)	0.181
Bleeding volume (ml)		1543.78 ± 1237.73	2450.00 ± 1746.42	1447.67 ± 1138.40	0.010	2028.00 ± 1474.30	1443.74 ± 1165.11	0.014
Volume of RBC (units)		4.36 ± 6.11	14.79 ± 9.58	3.26 ± 4.40	<0.001	9.16 ± 9.69	3.37 ± 4.53	0.008
Volume of plasma (ml)		738.38 ± 897.66	2000.00 ± 1619.12	604.57 ± 666.04	0.001	1416.00 ± 1399.90	598.37 ± 682.86	0.003
Volume of platelet (units)		2.32 ± 0.86	2.43 ± 1.45	2.30 ± 0.78	0.316	2.32 ± 1.11	2.31 ± 0.81	0.758
Use of norepinephrine		46 (31.51)	11 (7.53)	35 (23.97)	<0.001	14 (9.59)	32 (21.92)	0.004
Use of epinephrine		10 (6.85)	6 (4.11)	4 (2.74)	<0.001	7 (4.79)	3 (2.05)	<0.001
Use of dopamine		76 (52.05)	11(7.53)	65 (44.52)	0.037	18 (12.33)	58 (39.73)	0.028

### Mortality

#### Short-term mortality

The 30-day mortality was 9.59% (*n* = 14). Aged ≥ 50 years had a 14.732 (95%CI: 2.218–97.836) odds ratio of mortality as compared with < 50 years. The intraoperative volume of RBC was an independent risk factor for mortality, with odds ratio 1.262 (95%CI: 1.130–1.410). Intraoperative use of epinephrine had a 11.148 (95%CI: 1.738–71.525) odds of 30-day mortality as compared to without epinephrine (Table 2).

**Table 2:** Risk factors associated with 30-day mortality using multivariable logistic regression analysis

variables	Univariate Logistic regression				Multivariate Logistic regression			
	B	SE	OR(95%CI)	P value	B	SE	OR(95%CI)	P value
Age ≥ 50 (years)	1.267	0.574	3.552(1.152–10.948)	0.027	2.690	0.966	14.732(2.218–97.836)	0.005
Volume of RBC (units)	0.225	0.048	1.252(1.140–1.375)	<0.001	0.233	0.057	1.262(1.130–1.410)	<0.001
Use of epinephrine	3.178	0.741	24.000(5.614-102.608)	<0.001	2.411	0.948	11.148(1.738–71.525)	0.011

#### Long-term mortality

**Fig. 1:**
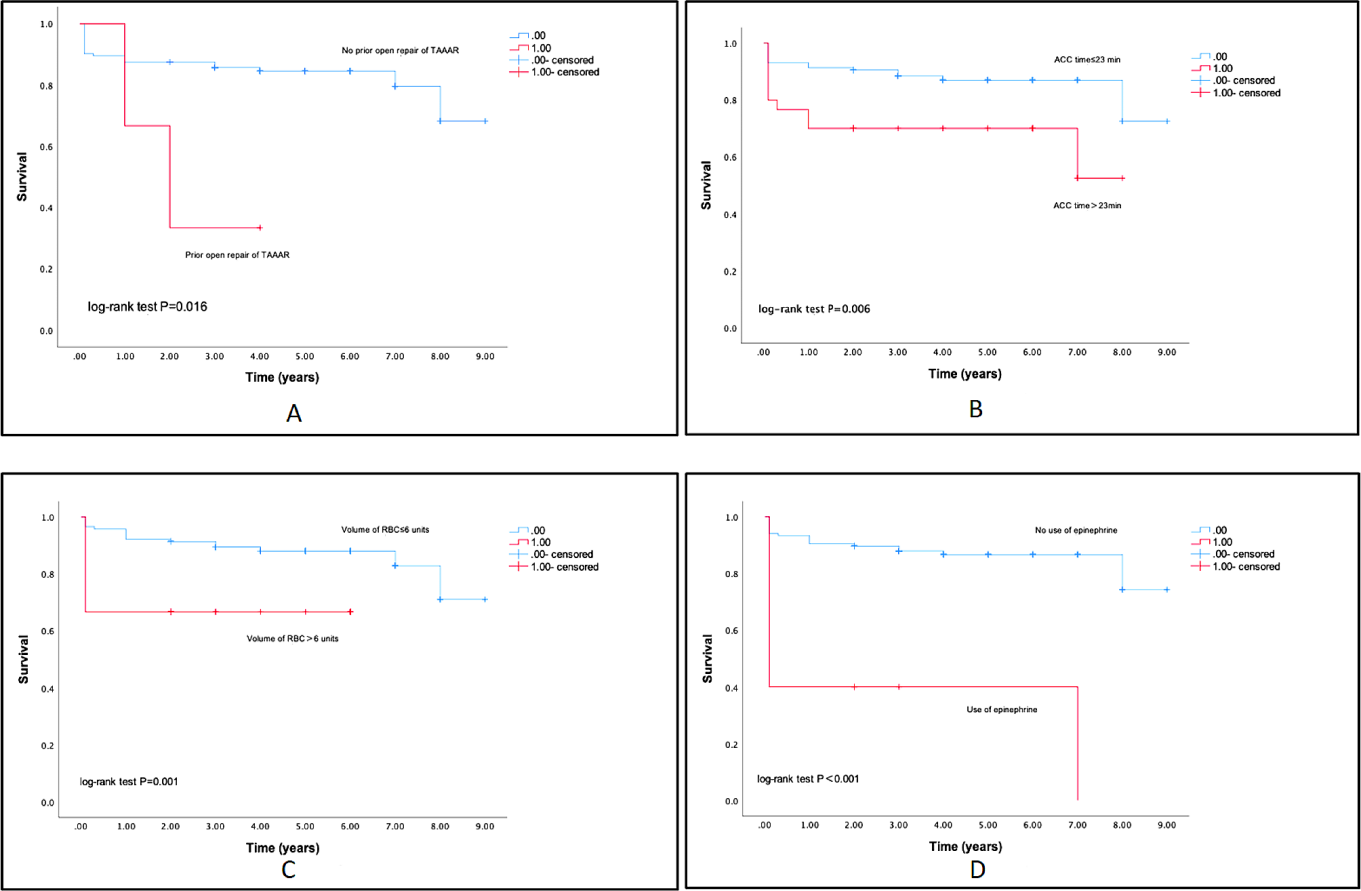
The Kaplan–Meier curves of the four independent risk factors: **(A)** Prior open repair of TAAAR (*n* = 2); **(B)** ACC time>23 min (*n* = 10); **(C)** Volume of RBC>6units (*n* = 13); **(D)** Use of epinephrine (*n* = 7)

The rate of follow-up was 85.62% (*n* = 125) and 14.38% (*n* = 21) was loss to follow-up due to changing contact information. Overall mortality was 17.12% (*n* = 25) at a median follow-up duration of 3.5 years (IQR = 2–5 years). Variables were further divided according to mortality status within 9 years between the two groups (Table 1). Patients with prior open TAAA repair had a 12.930 (95%CI: 2.722–61.427) hazard ratio of mortality as compared with those without (Table 3). Besides, the ACC time and intraoperative volume of RBC were independent risk factors for long-term mortality, with hazard ratio 1.016 (95%CI: 1.003–1.028) and 1.097 (95%CI: 1.004–1.152), respectively. Simultaneously, intraoperative use of epinephrine mortality had approximately four times hazard of mortality compared to without use of epinephrine. The Kaplan–Meier curves of the four independent risk factors evaluated by the Log-rank test are shown in Fig. 1. The cut points of ACC time is 23 min (IQR = 10–23 min, median = 17 min) and of intraoperative volume of RBC is 6 units (IQR = 0–6 units, median = 2 units).

**Table 3:** Risk factors associated with long-term mortality using multivariable Cox regression analysis

variables	Univariate Cox regression				Multivariate Cox regression			
	B	SE	HR(95%CI)	P value	B	SE	HR(95%CI)	P value
Prior open repair of TAAAR	1.563	0.741	4.771(1.116–20.391)	0.035	2.560	0.795	12.930(2.722–61.427)	0.001
ACC time (minutes)	0.016	0.005	1.016(1.005–1.027)	0.004	0.016	0.006	1.016(1.003–1.028)	0.014
Volume of RBC (units)	0.107	0.022	1.113(1.066–1.162)	<0.001	0.092	0.025	1.097(1.004–1.152)	<0.001
Use of epinephrine	2.057	0.459	7.820(3.180-19.233)	<0.001	1.393	0.484	4.026(1.558–10.405)	0.004

### Adverse outcomes

#### Short-term outcomes

The postoperative 30-day outcomes were presented in Table 4. Regarding to adverse complication, patients with stroke were 2.05% (*n* = 3), 13.70% (*n* = 20) patients suffered spinal cord injury, and patients suffering from respiratory complications, renal insufficiency and renal failure were 17.12% (*n* = 25), 18.49% (*n* = 27) and 19.18% (*n* = 28), respectively. In addition, intensive care unit (ICU) length of stay was 7.014 ± 8.674 days.

**Table 4:** 30-day postoperative outcomes

Outcomes	Numbers (%)
Conscious disturbance	6 (4.11)
Stroke	3 (2.05)
Spinal cord injury	20 (13.70)
Paraplegia	7 (4.79)
Paraparesis	13 (8.90)
Respiratory complications	25 (17.12)
Atrial fibrillation	7 (4.79)
Ventricular arrhythmia	14 (9.59)
Myocardial infarction	5 (3.42)
Renal insufficiency	27 (18.49)
Renal failure	28 (19.18)
Hypohepatia	108 (73.97)
Mechanical ventilation time	52.897 ± 72.508
ICU length of stay	7.014 ± 8.674
Wound infection	7 (4.79)
Secondary endotracheal Intubation	13 (8.90)
Secondary operation	21 (14.38)
Mortality	14 (9.59)

ICU, intensive care unit

#### Long-term outcomes

The postoperative long-term outcomes were listed in Table 5. At a 9-year follow-up, the overall incidence of stroke, permanent spinal cord injury, myocardial infarction (MI), renal failure, sexual dysfunction and permanent hoarseness was 5.48% (*n* = 8), 13.01% (*n* = 19), 3.42% (*n* = 5), 21.23% (*n* = 31), 2.74% (*n* = 4) and 1.37% (*n* = 2), respectively.

**Table 5:** long-term outcomes

Outcomes	Numbers (%)
Stroke	8 (5.48)
Permanent SCI	19 (13.01)
Immediate SCI	13 (8.90)
Delayed SCI	6 (4.11)
Myocardial infarction	5 (3.42)
Renal failure	31 (21.23)
Sexual dysfunction	4 (2.74)
Permanent hoarseness	2 (1.37)
Mortality	25 (17.12)

## Discussion

This study identified independent perioperative risk factors for both early and long term mortality after TAAA.

The 30-day mortality of 9.59% was higher than other research, as compared to 7–9% [4, 11–13]. However, this study only included extent II and extent III, and previous studies have shown that patients in extent II and III had increased rates of mortality (9.1–9.5% and 8.8%-12.7% respectively) and adverse outcomes [5, 9]. Three independent risk factors (aged ≥ 50 yeas, intraoperative volume of RBC, and intraoperative use of epinephrine) were associated with an increased risk for 30-day mortality. The incidence of mortality was increased obviously in the advanced aged population, which was also supported by previous studies from Anand Dayama et al. [14] and Joseph S. Coselli et al. [5] In this study we found that the intraoperative volume of RBC transfusion for patients with and without 30-day mortality are 14.79 ± 9.58 and 3.26 ± 4.40 units respectively. Increased intraoperative volume of RBC was an independent risk factor of 30-day mortality. Recent evaluations suggest that in the majority of clinical settings, restrictive RBC transfusion strategies are safe compared with liberal strategies which might potentially lead to adverse outcomes [15–17]. In 2009, M D Kertai et al. found that 30-day mortality of TAAA surgery was increased in patients receiving intraoperative RBC compared to those without (1 or 2 units of RBCs) [13]. Intraoperative use of RBC in patients with open repair of aortic aneurysm was independently related with perioperative mortality [13], which was also consistent with our study. According to the results presented above, it is advisable to consider more restrictive strategies concerning RBC transfusions for patients undergoing elective TAAA repair. Administering RBC transfusions at a conservative hemoglobin level of 7–8 g/dL might be considered viable without concerning about potential adverse effects [16]. Epinephrine is crucial for increasing cerebral and coronary perfusion pressure during circulatory instability [18]. In our research, out of 10 patients (6.85%) were applied 7258 ± 14,515 µg epinephrine, 6 (4.11%) of them who were received 11,408 ± 18,091 µg epinephrine experienced mortality within 30 days. The mortality and severe complications after TAAA repair are associated with hypoperfusion [19, 20].

Long-term survival rate was 82.88% (median of 3.5 years of follow-up). Multivariate Cox regression analysis showed four independent risk factors (prior open repair of aortic aneurysm, ACC time, increased intraoperative volume of RBC, intraoperative use of epinephrine) associated with an increased risk for long-term mortality. In this study we knew that the intraoperative volume of RBC transfusion for patients with and without Long-term death are 9.16 ± 9.69 and 3.37 ± 4.53 units respectively. Increased intraoperative volume of RBC and intraoperative use of epinephrine were independent risk factors for both early and long term mortality. In previous study, The transfusion of 4 or more units of RBC correlated with heightened mortality, and this elevated risk was maintained during the 15-year follow-up period [15]. In this research, 7 (4.79%) patients who were received 9850 ± 17,021 µg epinephrine experienced long-term mortality. Christopher J. R et al. research showed that epinephrine may lead to worse microcirculatory flow, and was associated with worse long-term outcomes [18]. Joseph V et al. presented that there was no significant association between prior TAAA repair and mortality of reoperative TAAA surgery [21]. However, we found that prior open TAAA repair was an independent risk factor for long-term mortality. In this study, the number of patients with prior TAAA repair (2.05%, *n* = 3) are fewer, compared with 7% (*n* = 20) [21], and the result may require a larger sample size to support. Furthermore, several previous studies [9, 12, 22], illustrated that increased ACC time was a risk factor for long-term mortality and adverse events. In our research, the intraoperative ACC time for patients with and without Long-term death are 34.88 ± 32.75 min and 19.69 ± 21.07 min respectively. An extensive ACC duration is characterized as significant when exceeding 30 min for TAAA surgery [22].

SCI is one of the serious complications after TAAAR, which can result in paraplegia and paraparesis [5, 9]. In this study, the incidence of immediate postoperative SCI is 13.70% (*n* = 20), with 8.90% of paraplegia and 4.79% of paraparesis. Seven cases were cured before discharge under active treatments. 6 cases were delayed SCI (SCI occurred after discharge from the hospital). The incidence of permanent SCI is 13.01% (*n* = 19). Surprisingly, in the process of follow-up, we found that four cases occurred sexual dysfunction after the surgery. In male, sexual dysfunction can result from erectile dysfunction (ED), which is caused by the inability to achieve or maintain an erection or ejaculation disorders [23, 24]. Previous clinical study showed that abdominal aortic aneurysm (AAA) surgical repair has an impact on sexual function which is caused by the involvement of the common iliac artery by AAA repair [23]. However, up to now, studies of sexual dysfunction after TAAAR are rare. TAAAR also involves the common iliac artery, so the occurrence of sexual function after such procedure requires more attention. Two patients happened permanent hoarseness after the surgery, and this outcome was generally associated with prolonged tracheal intubation [25–27].

Several approaches have been devised and are applied in surgery to minimize spinal cord injury, enhance spinal cord metabolism, and reduce ischemia in distal organs. These encompass profound hypothermic circulation arrest, intraoperative distal aortic perfusion, left heart bypass, monitoring spinal motor neurons through motor evoked potentials, cerebrospinal fluid drainage, and facilitating intraoperative spinal cord perfusion to reduce ischemia-reperfusion damage.

The primary limitation of current study is that the number of cases involved is small due to the difficulty of therapy and expensive cost. Moreover, this is a single-center study which is appropriate for Asian populations and the applicability in other populations require further researches to verify.

## Conclusion

Our study identified perioperative predictors for early and long term mortality after TAAA. This result could be instructive for perioperative patient management. In addition, the early and long term postoperative outcomes were presented. Although open repair of thoracoabdominal aortic aneurysm was the primary treatment method for patient at risk for lethal aneurysm rupture, the challenging mortality and adverse outcomes cannot be ignored.

## Data Availability

Data supporting the results reported in the article can be accessed by connecting wangyuefu@hotmail.com.

## Abbreviations

TAAA: Thoracoabdominal aortic aneurysm
RBC: Red blood cell
ACC: aortic cross-clamping
TAAAR: Repair of thoracoabdominal aortic aneurysm
SCI: spinal cord injury
DHCA: deep hypothermic circulatory arrest
ALT: Alanine aminotransferase
TBil: Total bilirubin
LDH: Lactic dehydrogenase
ARDS: Acute respiratory distress syndrome
IQR: Interquartile range
BMI: Body mass index
ICU: Intensive care unit
MI: Myocardial infarction
ED: Erectile dysfunction
AAA: Abdominal aortic aneurysm
